# Effects of Multi-Walled Carbon Nanotubes and Nano-Silica on Root Development, Leaf Photosynthesis, Active Oxygen and Nitrogen Metabolism in Maize

**DOI:** 10.3390/plants12081604

**Published:** 2023-04-10

**Authors:** Yubo Hao, Yang Yu, Guangyan Sun, Xiujie Gong, Yubo Jiang, Guoyi Lv, Yiteng Zhang, Liang Li, Yang Zhao, Dan Sun, Wanrong Gu, Chunrong Qian

**Affiliations:** 1 Institute of Crop Cultivation and Tillage, Heilongjiang Academy of Agricultural Sciences, Harbin 150028, China; 2 College of Agriculture, Northeast Agricultural University, Harbin 150030, China; 3 Institute of Crop Resource, Heilongjiang Academy of Agricultural Sciences, Harbin 150086, China

**Keywords:** maize, multi-walled carbon nanotubes, nano-silica

## Abstract

Carbon nanotubes (MWCNTs) and nano-silica (nano-SiO_2_) are widely used in the field of life science because of their special physical and chemical properties. In this study, the effects of different concentrations of MWCNTs (0 mg·L^−1^, 200 mg·L^−1^, 400 mg·L^−1^, 800 mg·L^−1^ and 1200 mg·L^−1^) and nano-SiO_2_ (0 mg·L^−1^, 150 mg·L^−1^, 800 mg·L^−1^, 1500 mg·L^−1^ and 2500 mg·L^−1^) on maize seedling growth and relative mechanisms were explored. The main results are as follows: MWCNTs and nano-SiO_2_ can promote the growth of maize seedlings, and promote plant height, root length, the dry and fresh weight of seedlings, root–shoot ratio and so on. The ability to accumulate dry matter increased, the relative water content of leaves increased, the electrical conductivity of leaves decreased, the stability of cell membranes improved and the water metabolism ability of maize seedlings increased. The treatment of MWCNTs with 800 mg·L^−1^ and nano-SiO_2_ with 1500 mg·L^−1^ had the best effect on seedling growth. MWCNTs and nano-SiO_2_ can promote the development of root morphology, increase root length, root surface area, average diameter, root volume and total root tip number and improve root activity, so as to improve the absorption capacity of roots to water and nutrition. After MWCNT and nano-SiO_2_ treatment, compared with the control, the contents of O_2_·^−^ and H_2_O_2_ decreased, and the damage of reactive oxygen free radicals to cells decreased. MWCNTs and nano-SiO_2_ can promote the clearance of reactive oxygen species and maintain the complete structure of cells, so as to slow down plant aging. The promoting effect of MWCNTs treated with 800 mg·L^−1^ and nano-SiO_2_ treated with 1500 mg·L^−1^ had the best effect. After treatment with MWCNTs and nano-SiO_2_, the activities of key photosynthesis enzymes PEPC, Rubisco, NADP-ME, NADP-MDH and PPDK of maize seedlings increased, which promoted the opening of stomata, improved the fixation efficiency of CO_2_, improved the photosynthetic process of maize plants and promoted plant growth. The promoting effect was the best when the concentration of MWCNTs was 800 mg·L^−1^ and the concentration of nano-SiO_2_ was 1500 mg·L^−1^. MWCNTs and nano-SiO_2_ can increase the activities of the enzymes GS, GOGAT, GAD and GDH related to nitrogen metabolism in maize leaves and roots, and can increase the content of pyruvate, so as to promote the synthesis of carbohydrates and the utilization of nitrogen and promote plant growth.

## 1. Introduction

With the development of China’s economy and the improvement of people’s living standards, a high and stable yield of maize is imminent, and it is urgent to find new agricultural technologies and products [1,2,3,4]. The rise of nanomaterials in the 1980s has attracted wide attention from scientific researchers and explored the possibility of their application in various areas [2]. With the continuous optimization of the manufacturing process of nano-materials, nano-materials have shown significant effects in the agricultural field. They are increasingly widely used in agricultural production and have outstanding prospects in improving production efficiency, such as nano-pesticide, nano-fertilizer, soil passivator, etc. Research shows that nano-materials can improve crop stress resistance and protect them from pests, improve crop photosynthesis efficiency and root nutrient absorption and water transport capacity and improve crop yield and quality, thereby reducing fertilizer application [3,4]. In the agricultural production process, compared with the traditional formula, the use of nano formula pesticides and other nano formula agricultural chemicals can improve their efficacy, which can be achieved by reducing the size of active ingredients and other compounds to the nanometer range, mixing them into nano lotion or nano dispersions, or adding them to solid lipids or polymer nanocapsules.

Several metallic and non-metallic nanoparticles, such as silver nanoparticles, gold nanoparticles, copper nanoparticles, iron nanoparticles, titanium dioxide nanoparticles, zinc and zinc oxide nanoparticles, carbon-based nanoparticles and fullerenes, have been widely used to promote the pretreatment of seed germination, seedling growth, improve absorption and enhance the exchange between plants, the environment and crop stress tolerance [5]. Studies on lettuce and cucumber, mustard, frankincense and galan have shown that nano-Au can promote seedling growth and increase plant height, chlorophyll and sugar content [6,7,8,9]. Applying nano-SiO_2_ can promote the development of Larix olgensis seedlings, improve the seedling height and root growth-related indicators, and is conducive to plant photosynthesis [10]. Under drought stress, nano-TiO_2_ promoted wheat plants’ growth and development and yield [11]. In addition, nano-TiO_2_ can also promote the growth of rape seedlings, and the root length and bud length are improved [12]. Research shows that nano-TiO_2_ can enhance the activity of enzymes related to nitrogen metabolism and promote the conversion of inorganic nitrogen into organic nitrogen in plants, which is conducive to the increased plant biomass [13]. Applying nano-TiO_2_ or nano-ZnO can promote lettuce to absorb N, P, K, Zn and other nutrients, which is an effective way to improve lettuce yield [14]. Nano-ZnO can be absorbed by the roots of mungbean and chickpea seedlings, which increases the length of rhizome and its dry and fresh weight [15]. Wheat plants can absorb water-soluble CNTs, which shows that CNTs can promote the growth and development of plants mainly because they enhance water absorption and nutrients [16,17,18,19]. Due to its special chemical characteristics, nano-TiO_2_ can be used as a photocatalyst for catalytic oxidation and reduction reactions [20], which can improve the absorption of light by plants and enhance the efficiency of photosynthesis. Research shows that nano-TiO_2_ can promote the increase in the net photosynthetic rate and transpiration rate of crops [3]. In addition, nano-TiO_2_ can also significantly improve the vitality of aging seeds, protect chloroplasts, promote the formation of chlorophyll [21], improve the Rubisco enzyme activity and photosynthetic efficiency and promote crop growth [13,22]. This may be because nano-TiO_2_ can enhance the activity of a Rubisco activating enzyme and its protein level [23]. In tomatoes and pumpkins under NaCl stress, nano-SiO_2_ can stimulate their antioxidant systems and alleviate the damage caused by stress [24,25]. Research shows that nano-Si can reduce the accumulation of metal Cr in pea seedlings and the content of active oxygen in plants [26,27].

Carbon nanotubes are cylindrical structures with a diameter of several nanometers composed of rolled graphene sheets. According to the structure of carbon nanotubes, they can be divided into single-wall nanotubes (SWCNT) and multi-wall nanotubes (MWCNTs). Multi-walled carbon nanotubes have a 5–40 nm diameter and a length of about 10 μm [28]. In addition to the positive effects of multi-wall carbon nanotubes on seed germination, there are many reports that the effects of multi-wall carbon nanotubes have not been found in many different plant species, including radish, rape, ryegrass, lettuce, maize, wheat [29], mustard, black lentil and zucchini [30]. This difference may be attributed to the genotype difference of the tested seed material or the variation of the seed batch quality, but the test conditions may also cause it. A study on the effect of multi-wall carbon nanotubes on the plant cell culture of Arabidopsis thaliana suspension cells showed that multi-wall carbon nanotubes (10–600 mg·L^−1^) had toxic effects [31]. By inhibiting cell growth and cell activity, chlorophyll content and superoxide dismutase (SOD) activity decreased. The direct cell contact of single or aggregated carbon nanotubes seems to be the prerequisite for inducing plants to produce positive or adverse reactions. The difference of plant sensitivity and different physical parameters of carbon nanotubes (diameter, length and aggregation degree) is the decisive factor for expressing plant response variables. Compared with the variants containing larger aggregates of the same multi-walled carbon nanotubes, the high dispersion of multi-walled carbon nanotubes in the growth medium leads to a more substantial stimulus to plant growth [19], which indicates that the uniform and widely distributed contact between the smaller multi-walled carbon nanotubes and plant tissue may be a prerequisite for promoting plant growth. In many studies, the expression of the effect depends on the concentration. It has a positive effect in applying multi-walled carbon nanotubes at lower concentrations, and has an inhibitory effect at higher concentrations [30,32]. Oxidative stress induction related to the formation of reactive oxygen species, membrane damage, electrolyte leakage, mitochondrial dysfunction, DNA distortion and cell death has been identified as the determinant of the toxicity of multi-walled carbon nanotubes. The above research results show that carbon nanotubes are a stress factor that can induce plant defense response and excitatory effect or toxicity, which depends on the stimulation intensity.

Silicon dioxide (SiO_2_) mainly exists in the form of crystal in nature and is composed of SiO_4_ units arranged in a tetrahedral geometry, such as sand, quartz, etc. Compared with the naturally occurring silica, nano-SiO_2_ is amorphous in nature, and its size is mainly between 5 and 100 nm [33]. Compared with bulk silica, nano-silica has some advantages, such as a relatively high surface area to volume ratio, unique thermal and electrical properties and increased permeability in plant cells [34]. These nanomaterials are of great significance in the fields of agriculture and biomedicine. Nano-SiO_2_ enhanced seed germination and stimulated the antioxidant system under NaCl stress [35]. The exogenous application of nano-SiO_2_ and nano-TiO_2_ improves the germination rate of soybean seeds by increasing nitrate reductase [36] and by enhancing the ability of seeds to absorb and use water and nutrients [37]. Under salt stress, nano-SiO_2_ increased the fresh weight, dry weight, chlorophyll content and proline accumulation of leaves. Nano-SiO_2_ increases proline, free amino acid, nutrient content and antioxidant enzyme activity, thus improving the tolerance of plants to abiotic stress [24,25,38]. Nano-SiO_2_ promotes plant growth and development by increasing gas exchange and chlorophyll fluorescence parameters [25]. Nanoparticles have deep toxic effects on plant systems and food crops, and the toxicity depends on various factors, such as size, concentration, stability and synthesis process [39,40]. The phytotoxic effect of nano-SiO_2_ has also been reported [41,42]. Low concentrations (<200 mg·L^−1^) of nano-silica can promote plant growth, while high concentrations of nano-silica will affect the growth of wheat plants and lead to lower chlorophyll content, lipid peroxidation and higher antioxidant enzyme activity of wheat [43]. Among them, when Bt transgenic cotton was treated with a high dose of nano-SiO_2_, the biomass of root and shoot decreased significantly [44].

Current research on nanomaterials mainly includes research on using nanomaterials as new fertilizers to increase crop production, as well as exploring the possibility of combining nanomaterials with agriculture and ecological security. In addition, most current research is focused on the impact of nanomaterials on a single stage of plant growth, while relatively few studies have investigated whether nanomaterials have different effects on different stages of plant growth. Therefore, exploring the response mechanism of maize to nanomaterials and the correlation between nanomaterial concentration and impact effects is a very important research issue that is crucial for understanding the biological effect mechanism of nanomaterials on maize growth and development. In this study, the biomass, root morphology, key enzymes of photosynthesis, nitrogen metabolism index, antioxidant index, etc., of maize seedlings under different concentrations of MWCNTs and nano-SiO_2_ were measured through hydroponic experiments, and the response mechanism of maize to nano-materials and the correlation between the concentration of nano-materials and the effect were explored. The research provides a theoretical and experimental basis for applying the application of multi-wall carbon nanotubes and nano-silica in agriculture.

## 2. Results

### 2.1. Seedling Morphology

**Figure 1:**
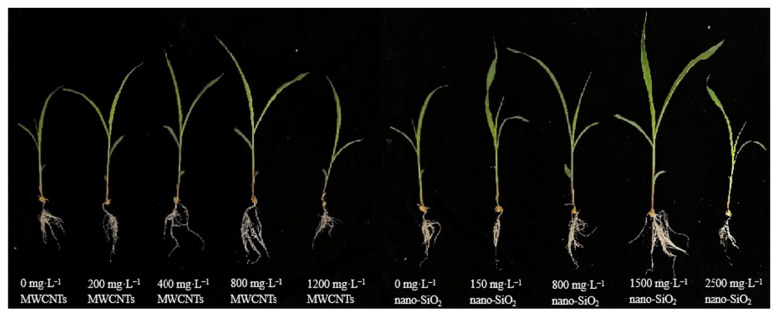
Effect of different concentrations of MWCNTs and nano-SiO_2_ on maize seedling morphology.

It can be seen from Figure 1 that the treatment of MWCNTs and nano-SiO_2_ with different concentrations promoted the growth of maize seedlings in varying degrees. The plant height of maize seedlings increased, the leaves were darker, the roots grew and the growth was robust. At the same time, with the increase in the concentration, the promotional effect showed a trend of first strengthening and then weakening. MWCNTs had the best promotional effect at a concentration of 800 mg·L^−1^ and that of nano-SiO_2_ at a concentration of 1500 mg·L^−1^ (Figure 1).

### 2.2. Dry and Fresh Weight of Seedlings

It can be seen from Table 1 that different concentrations of MWCNTs and nano-SiO_2_ promote the growth of maize seedlings, and promote the height of maize seedlings, the fresh weight, dry weight and root–shoot ratio of seedlings and roots. MWCNTs at 800 mg·L^−1^ and nano-SiO_2_ at 1500 mg·L^−1^ have the most significant lifting effect. At the same time, with the increase in the concentration of the solution, the promotional effect showed a trend of first strengthening and then weakening. Different concentrations of MWCNTs significantly increased the height of maize seedlings, 11.09%, 33.43%, 42.22% and 16.73%, respectively, compared with the control. Nano-SiO_2_ at 800 mg·L^−1^ and 1500 mg·L^−1^ significantly increased the height of maize seedlings, 32.62% and 46.74% higher than that of the control, respectively. The other treatments had no significant difference compared with the control. MWCNTs of 800 mg·L^−1^ and nano-SiO_2_ of 1500 mg·L^−1^ significantly increased the fresh weight of maize seedlings, 39.82% and 43.34% higher than that of the control, respectively. However, the promotional effect of other treatments did not reach a significant level. Different concentrations of MWCNTs increased the fresh root weight of maize seedlings, which were 31.33%, 45.98%, 69.43% and 40.67% higher than the control, respectively. The promotional effect of low concentration treatment on the fresh root weight gradually increased, while the promotional effect of high concentration treatment on the fresh root weight decreased. Compared with the control, the fresh weight of maize seedling roots increased by 19.86%, 39.00%, 62.77% and 25.72% with different concentrations of nano-SiO_2_. Different concentrations of MWCNTs significantly increased the root–shoot ratio of maize seedlings, 14.44%, 17.33%, 22.49% and 16.35% higher than that of the control, respectively, but there was no significant difference between the treatments. The root–shoot ratio of maize seedlings was significantly increased at the concentration of nano-SiO_2_ at 800 mg·L^−1^ and 1500 mg·L^−1^, which were 12.27% and 14.94% higher than that of the control, respectively. There was no significant difference between the low and high concentrations (150 mg·L^−1^ and 2500 mg·L^−1^) and the control. The treatment of MWCNTs with 400 mg·L^−1^ and 800 mg·L^−1^ and nano-SiO_2_ with 800 mg·L^−1^ and 1500 mg·L^−1^ significantly increased the dry weight of maize seedling root and seedling, which increased the dry weight of seedling root by 23.53%, 33.14%, 29.60% and 43.52%, respectively, compared with the control, and increased the dry weight of seedling by 52.07%, 90.91%, 58.68% and 78.93%, respectively, while the effect of other treatments did not reach a significant level (Table 1).

### 2.3. Relative Water Content of Leaves

**Figure 2:**
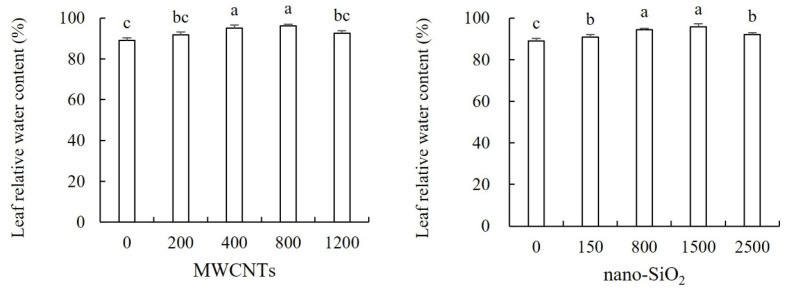
Effect of different concentrations of MWCNTs and nano-SiO_2_ on relative water content in maize. Note: Data are expressed as mean ± standard deviation. Different letters within the same column indicate significant difference at 5% level.

The effects of different concentrations of MWCNTs and nano-SiO_2_ on the relative water content of maize leaves are shown in Figure 2. Both of them increased the relative water content of maize leaves to varying degrees, and with the increase in their concentrations, their promoting effects first increased and then decreased, of which 800 mg·L^−1^ MWCNTs and 1500 mg·L^−1^ nano-SiO_2_ had the best effect. In the treatment of MWCNTs, the moderate concentration of MWCNTs (400 mg·L^−1^ and 800 mg·L^−1^) significantly increased the relative water content of maize leaves by 5.81% and 7.95%, respectively, compared with the control. There was no significant difference between the other treatments and the control. In the treatment of nano-SiO_2_, the relative water content of maize leaves was significantly increased by 2.03%, 6.11%, 7.63% and 3.52% compared with the control (Figure 2).

### 2.4. Electrical Conductivity of Leaves

**Figure 3:**
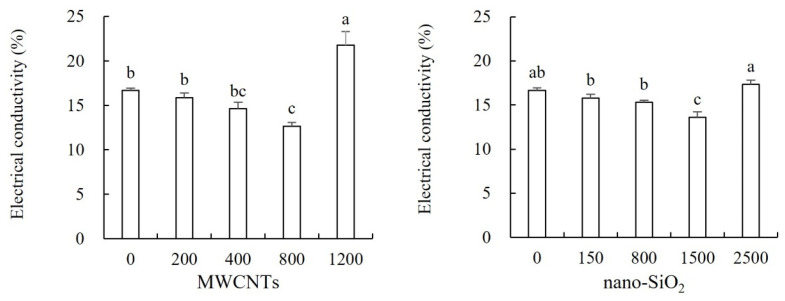
Effect of different concentrations of MWCNTs and nano-SiO_2_ on electrical conductivity in maize. Note: Data are expressed as mean ± standard deviation. Different letters within the same column indicate significant difference at 5% level.

As can be seen from Figure 3, lower concentrations of MWCNTs and nano-SiO_2_ decreased the relative conductivity of maize leaves, while higher concentrations increased the relative conductivity of maize leaves. In the treatment of MWCNTs, lower concentrations of MWCNTs (200 mg·L^−1^, 400 mg·L^−1^ and 800 mg·L^−1^) decreased the relative conductivity of maize leaves by 4.80%, 12.33% and 24.11% compared with the control, and the treatment of 800 mg·L^−1^ MWCNTs was the best. MWCNTs of 1200 mg·L^−1^ significantly increased the relative conductivity of maize leaves, which was 30.65% higher than that of the control. Compared with the control, the relative conductivity of maize leaves decreased by 5.24%, 8.16% and 18.30% in the treatment of low concentrations of nano-SiO_2_ (150 mg·L^−1^, 800 mg·L^−1^ and 1500 mg·L^−1^), and the treatment of 1500 mg·L^−1^ nano-SiO_2_ had the best effect. Compared with the control, the increase in the relative conductivity of maize leaves by high concentration of nano-SiO_2_ (2500 mg·L^−1^) did not reach a significant level (Figure 3).

### 2.5. Root Morphology

As shown in Table 2, different concentrations of MWCNTs and nano-SiO_2_ both increased the root length, root surface area, root volume and total root tip number of maize seedlings. With the increase in solution concentration, the promotional effect first increased and then decreased. MWCNTs of 800 mg·L^−1^ and nano-SiO_2_ of 1500 mg·L^−1^ had the best effect, and the promotional effect of MWCNTs was slightly stronger than that of nano-SiO_2_. The MWCNTs of 400 mg·L^−1^ and 800 mg·L^−1^ significantly increased the root length, root surface area and total root tip number of maize seedlings, in which the root length increased by 66.13% and 98.09%, respectively, the root surface area increased by 30.44% and 56.62%, respectively, and the total root tip number increased by 676.24% and 1243.65%, respectively, compared with the control. The promotional effect of other treatments was not significantly different from that of the control. The root volume increased by 13.45%, 52.38%, 85.43% and 29.13%, respectively, compared with the control under the treatment of different concentrations of MWCNTs, of which the promotional effect of only 800 mg·L^−1^ MWCNTs reached a significant level, and there was no significant difference between the other treatments and the control. The higher concentration of nano-SiO_2_ (800 mg·L^−1^, 1500 mg·L^−1^ and 2500 mg·L^−1^) significantly increased the root length and root surface area of maize seedlings, among which the root length increased by 41.00%, 125.97% and 57.85%, respectively, compared with the control, and the root surface area increased by 34.27%, 68.20% and 43.62%, respectively, compared with the control, while the improvement effect of other treatments did not reach a significant level. The root volume increased by 50.70%, 94.96% and 11.48%, respectively, under the treatment of nano-SiO_2_ with different concentrations, of which only 1500 mg·L^−1^ nano-SiO_2_ reached a significant level. Nano-SiO_2_ at 800 mg·L^−1^ and 1500 mg·L^−1^ significantly increased the total root tip number of maize seedlings, which were 191.71% and 1201.66% higher than that of the control, respectively. The average root diameter is the average value of the sum of all root diameters. Its value cannot directly reflect the quality of root development. It is necessary to combine the number of roots and root scanning pictures. The average root diameter of MWCNTs treated with 400 mg·L^−1^ and 800 mg·L^−1^ was 26.12%, 39.24% and 5.76% lower than that of the control, and that of nano-SiO_2_ treated with 800 mg·L^−1^, 1500 mg·L^−1^ and 2500 mg·L^−1^ was 33.99% and 42.63% lower than that of the control, respectively. MWCNTs of 200 mg·L^−1^, 1200 mg·L^−1^ and nano-SiO_2_ of 150 mg·L^−1^ increased the average root diameter by 26.76%, 11.74% and 5.98%, respectively, compared with the control, but there was no significant difference compared with the control (Table 2).

### 2.6. Root Activity

**Figure 4:**
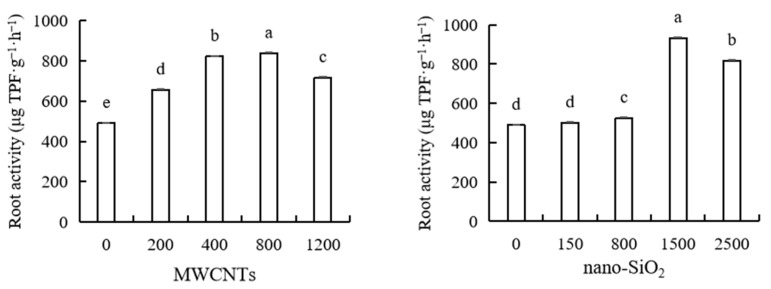
Effect of different concentrations of MWCNTs and nano-SiO_2_ on root activity in maize. Note: Data are expressed as mean ± standard deviation. Different letters within the same column indicate significant difference at 5% level.

The results showed that different concentrations of MWCNTs and nano-SiO_2_ had a promoting effect on maize root activity, and the promoting effect first increased and then decreased with the increase in MWCNTs and nano-SiO_2_ concentrations. The best promoting concentration of MWCNTs on maize root activity was 800 mg·L^−1^, and the best promoting concentration of nano-SiO_2_ was 1500 mg·L^−1^. Different concentrations of MWCNTs significantly increased the root activity of maize by 33.97%, 67.88%, 70.95%, and 46.28%, respectively, compared with the control. The root activity of maize was significantly increased by 7.28%, 90.63% and 66.84% compared with the control under the treatment of higher concentrations of nano-SiO_2_ (800 mg·L^−1^, 1500 mg·L^−1^ and 2500 mg·L^−1^), but the promotional effect of a low concentration of nano-SiO_2_ (150 mg·L^−1^) was not significantly different from that of the control (Figure 4).

### 2.7. Detection of Superoxide Anion (O_2_·^−^) and Hydrogen Peroxide (H_2_O_2_) in Leaves

**Figure 5:**
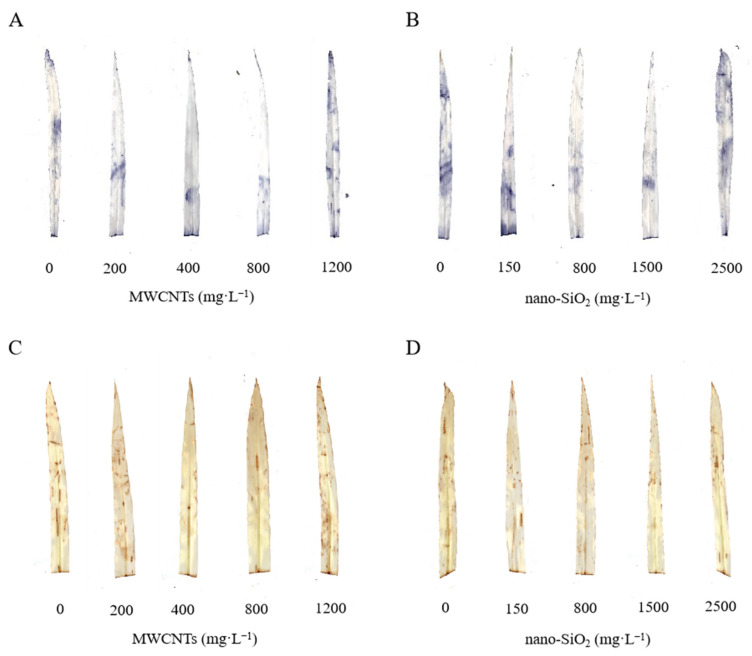
Effect of different concentrations of MWCNTs and nano-SiO_2_ on detection of superoxide anion (O_2_·^−^) (**A**,**B**) and hydrogen peroxide (H2O2) (**C**,**D**) in vivo in maize leaves.

It can be seen from Figure 5 that different concentrations of MWCNTs and nano-SiO_2_ both increase the rate of O_2_·^−^ formation and the content of H_2_O_2_ in maize leaves, and their lifting effect gradually decreases with the increase in concentration, of which the optimal concentration of MWCNTs is 800 mg·L^−1^, and the optimal concentration of nano-SiO_2_ is 1500 mg·L^−1^. After treatment with MWCNTs and nano-SiO_2_, maize leaves distributed less dark blue (which can represent the distribution and content of O_2_·^−^) and brown spots (which can represent the distribution and content of H_2_O_2_) than those of the control, indicating that different concentrations of MWCNTs and nano-SiO_2_ can reduce the production of active oxygen in maize seedling leaves (Figure 5).

### 2.8. Activities of Phosphoenolpyruvate Carboxylase (PEPC), Ribose Diphosphate Carboxylase (Rubisco), Malase (NADP-ME), Fructose Dehydrogenase (NADP-MDH) and Pyruvate Phosphate Double Kinase (PPDK)

**Figure 6:**
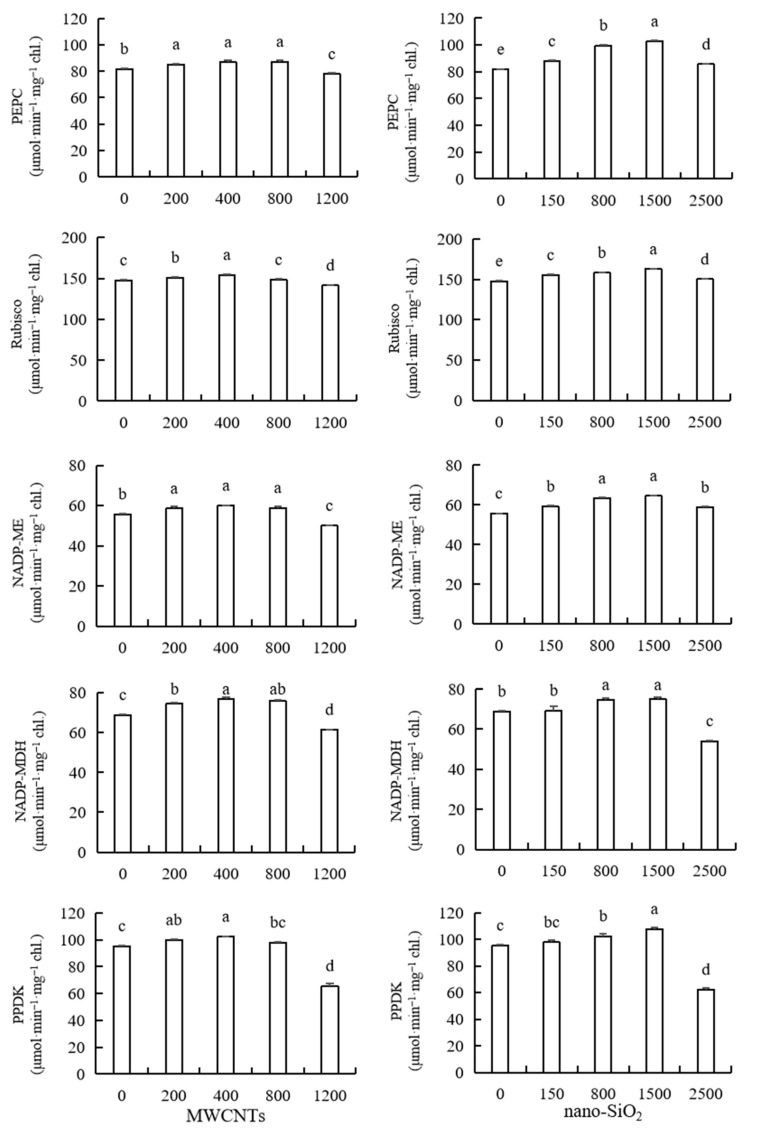
Effect of different concentrations of MWCNTs and nano-SiO_2_ on leaf photosynthetic enzymes in maize. Note: Data are expressed as mean ± standard deviation. Different letters within the same column indicate significant difference at 5% level.

It can be seen from Figure 6 that the lower concentration of MWCNTs promoted the photosynthetic enzyme activity of maize leaves (PEPC, Rubisco, NADP-ME, NADP-MDH and PPDK), while the higher concentration of MWCNTs inhibited the photosynthetic enzyme activity of maize leaves. PEPC enzyme activity was significantly increased by 4.24%, 6.29% and 6.60% compared with the control under the treatment of lower concentrations of MWCNTs (200 mg·L^−1^, 400 mg·L^−1^, 800 mg·L^−1^). Rubisco enzyme activity increased by 2.25%, 4.50% and 0.29% compared with the control under MWCNTs with concentrations of 200 mg·L^−1^, 400 mg·L^−1^ and 800 mg·L^−1^. MWCNTs of 200 mg·L^−1^, 400 mg·L^−1^ and 800 mg·L^−1^ significantly increased the activity of the NADP-ME enzyme, and there was no significant difference between the effects, which were 5.14%, 7.60%, and 5.55% higher than those of the control, respectively. NADP-MDH enzyme activity was significantly increased by 8.05%, 11.61% and 10.22% compared with the control under MWCNTs with concentrations of 200 mg·L^−1^, 400 mg·L^−1^ and 800 mg·L^−1^. The activity of the PPDK enzyme increased by 4.77%, 7.43% and 2.78%, respectively, compared with the control at lower concentrations. The activities of PEPC, Rubisco, NADP-ME, NADP-MDH and PPDK in maize leaves decreased significantly by 4.73%, 4.36%, 10.19%, 10.79%, 4.79% and 31.27%, respectively, under the treatment of a high concentration of MWCNTs (1200 mg·L^−1^). MWCNTs of 800 mg·L^−1^ have the best promotional effect on PEPC enzyme activity, while MWCNTs of 400 mg·L^−1^ have the best promotional effect on Rubisco, NADP-ME, NADP-MDH and PPDK enzyme activity (Figure 6).

Different concentrations of nano-SiO_2_ significantly increased the activities of PEPC, Rubisco and NADP-ME enzymes in maize leaves. With the increase in concentration, the promotional effect showed a trend of first increasing and then decreasing, among which the best promotional concentration was 1500 mg·L^−1^. The PEPC enzyme activity of maize leaves increased by 7.29%, 21.80%, 25.47% and 4.55%, respectively, compared with the control under different concentrations of nano-SiO_2_, and the Rubisco enzyme activity of maize leaves increased by 5.07%, 7.00%, 10.06% and 1.58%, respectively, compared with the control under its treatment, while the NADP-ME enzyme activity of maize leaves increased by 6.22%, 13.57%, 15.84% and 5.41%, respectively, compared with the control under its treatment. The lower concentrations of nano-SiO_2_ (150 mg·L^−1^, 800 mg·L^−1^, 1500 mg·L^−1^) promoted the activities of NADP-MDH and PPDK enzymes in maize leaves, and the optimum concentration was 1500 mg·L^−1^, while the higher concentration of nano-SiO_2_ (2500 mg·L^−1^) inhibited the activities of NADP-MDH and PPDK enzymes in maize leaves. Compared with the control, the NADP-MDH enzyme activity of maize leaves increased by 0.72%, 8.45% and 9.13%, respectively, under the treatment of low concentrations of nano-SiO_2_, and the PPDK enzyme activity of maize leaves increased by 2.45%, 7.23% and 12.92%, respectively, under the treatment. The activity of NADP-MDH and PPDK in maize leaves decreased by 21.50% and 34.80%, respectively, compared with the control under the treatment of a high concentration of nano-SiO_2_ (Figure 6).

### 2.9. Pyruvic Acid Content

**Figure 7:**
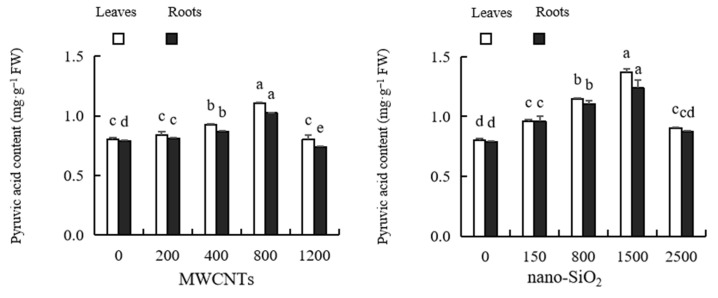
Effect of different concentrations of MWCNTs and nano-SiO_2_ on pyruvic acid content in maize. Note: Data are expressed as mean ± standard deviation. Different letters within the same column indicate significant difference at 5% level.

It can be seen from Figure 7 that with the increase in MWCNTs and nano-SiO_2_ concentrations, the pyruvic acid content in maize leaves and roots shows a trend of rising first and then falling. The optimum concentration of MWCNTs is 800 mg·L^−1^, and the optimum concentration of nano-SiO_2_ is 1500 mg·L^−1^. In the content of pyruvate in maize leaves, the promotion of MWCNTs of 400 mg·L^−1^ and 800 mg·L^−1^ reached significant levels, which were 15.59% and 38.01% higher than those of the control, respectively. The other treatments had no significant difference from the control. Low concentrations of MWCNTs significantly increased the pyruvic acid content in maize roots by 2.35%, 10.01% and 29.51%, respectively, compared with the control. A high concentration of MWCNTs (1200 mg·L^−1^) significantly reduced the pyruvic acid content in maize roots, which was 6.26% lower than that of the control. The promotion of nano-SiO_2_ at various concentrations on the pyruvate content in maize leaves reached a significant level, which was 19.66%, 43.48%, 71.12% and 12.98% higher than that of the control, respectively. The content of pyruvic acid in maize roots was significantly increased by low concentrations of nano-SiO_2_, which was 21.09%, 40.10% and 56.71% higher than that of the control, respectively. The promotional effect of a high concentration of nano-SiO_2_ was not significantly different from that of the control. The inhibitory effect of nano-SiO_2_ on pyruvate content in maize leaves and roots was slightly stronger than that of MWCNTs (Figure 7).

### 2.10. Glutamine Synthetase (GS) Activity

**Figure 8:**
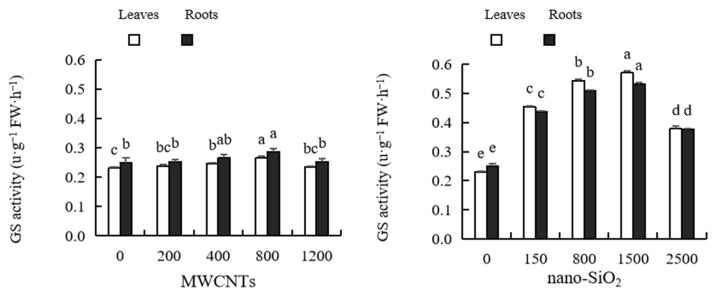
Effect of different concentrations of MWCNTs and nano-SiO_2_ on GS activity in maize. Note: Data are expressed as mean ± standard deviation. Different letters within the same column indicate significant difference at 5% level.

As shown in Figure 8, the activity of GS in maize leaves and roots first increased and then decreased with the increase in MWCNTs and nano-SiO_2_ concentrations, in which the optimal concentration of MWCNTs was 800 mg·L^−1^, and the optimal concentration of nano-SiO_2_ was 1500 mg·L^−1^. In the activity of GAD in maize leaves, after treatment with MWCNTs of different concentrations, it increased by 3.41%, 6.50%, 15.42% and 1.56% compared with the control, respectively. Among them, the promotion of 400 mg·L^−1^ and 800 mg·L^−1^ MWCNTs reached a significant level, and the other treatments had no significant difference with the control. The GAD activity of maize roots increased by 1.24%, 6.01%, 14.58% and 1.20%, respectively, compared with the control after treatment with MWCNTs of different concentrations, among which only MWCNTs of 800 mg·L^−1^ had a significant increase, and the rest had no significant difference compared with the control. After treatment with nano-SiO_2_ at various concentrations, the GS activity of maize leaves increased by 96.91%, 136.12%, 147.87% and 64.25%, respectively, compared with the control; the activity of GS in maize roots increased by 74.79%, 103.64%, 113.17% and 50.98%, respectively, compared with the control. At the same time, it can be seen that the promotional effect of nano-SiO_2_ on the GS activity of maize leaves and roots is stronger than that of MWCNTs (Figure 8).

### 2.11. Glutamic Acid Synthase (GOGAT) Activity

**Figure 9:**
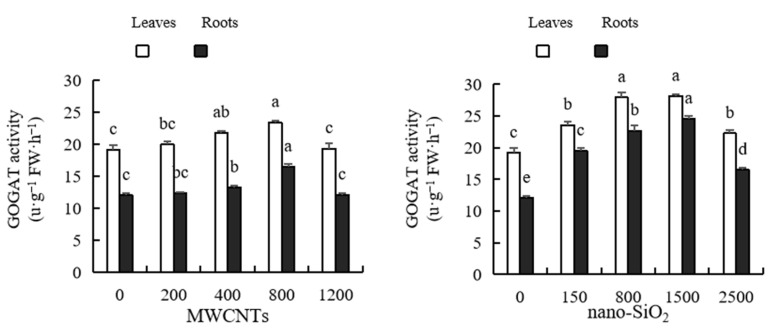
Effect of different concentrations of MWCNTs and nano-SiO_2_ on GOGAT activity in maize. Note: Data are expressed as mean ± standard deviation. Different letters within the same column indicate significant difference at 5% level.

As shown in Figure 9, with the increase in MWCNTs and nano-SiO_2_ concentrations, the GOGAT activity of maize leaves and roots first increased and then decreased, reaching the peak when the MWCNT concentration was 800 mg·L^−1^ and the nano-SiO_2_ concentration was 1500 mg·L^−1^, respectively. After treatment with MWCNTs of various concentrations, the GOGAT activity of maize leaves increased by 4.25%, 13.71%, 22.18% and 0.94%, respectively, compared with the control, and the GOGAT activity of maize roots increased by 2.75%, 10.12%, 36.90% and 0.48%, respectively, compared with the control, of which only the promotional effect of MWCNTs of 400 mg·L^−1^ and 800 mg·L^−1^ reached a significant level. The GOGAT activity of maize leaves treated with different concentrations of nano-SiO_2_ increased by 22.94%, 45.61%, 46.62% and 16.09%, respectively, compared with the control, of which only 800 mg·L^−1^ and 1500 mg·L^−1^ of nano-SiO_2_ had no significant difference. The GOGAT activity of maize roots increased by 61.80%, 88.44%, 103.81% and 36.75%, respectively, compared with the control after treatment with nano-SiO_2_ at various concentrations. The effect of nano-SiO_2_ on the GOGAT activity of maize leaves and roots was stronger than that of MWCNTs (Figure 9).

### 2.12. Glutamic Acid Decarboxylase (GAD) Activity

**Figure 10:**
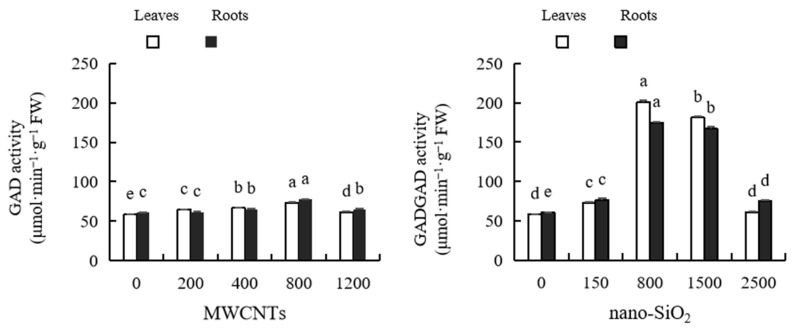
Effect of different concentrations of MWCNTs and nano-SiO_2_ on GAD activity in maize. Note: Data are expressed as mean ± standard deviation. Different letters within the same column indicate significant difference at 5% level.

As shown in Figure 10, different concentrations of MWCNTs and nano-SiO_2_ both increased the GAD activity of maize leaves and roots. With the increase in concentration, the GAD activity of maize leaves and roots first increased and then decreased. The optimum concentrations of MWCNTs and nano-SiO_2_ were 800 mg·L^−1^. In the activity of GAD in maize leaves, the promotional effect of MWCNTs at various concentrations reached a significant level, which was 11.10%, 14.30%, 26.10% and 5.78% higher than that of the control, respectively. After treatment with MWCNTs of various concentrations, the GAD activity of maize roots was 1.50%, 7.11%, 27.25% and 6.81% higher than that of the control, respectively. Among them, only 800 mg·L^−1^ MWCNTs did not reach a significant level, and the other treatments had significant differences compared with the control. In the activity of GAD in maize leaves, except for the treatment of nano-SiO_2_ with 2500 mg·L^−1^, there was no significant difference with the control, and the promotional effect of other concentration treatments reached a significant level, increasing by 24.98%, 245.73%, 211.62% and 5.10%, respectively, compared with the control. All concentrations of nano-SiO_2_ significantly increased the activity of GAD in maize roots by 25.65%, 188.14%, 175.57% and 28.53%, respectively, compared with the control. The promotional effect of nano-SiO_2_ on the GAD activity of maize leaves and roots is stronger than that of MWCNTs (Figure 10).

### 2.13. Glutamic Acid Dehydrogenase (GDH) Activity

**Figure 11:**
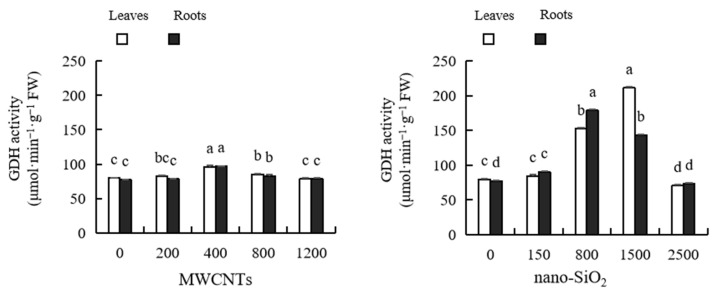
Effect of different concentrations of MWCNTs and nano-SiO_2_ on GDH activity in maize. Note: Data are expressed as mean ± standard deviation. Different letters within the same column indicate significant difference at 5% level.

As shown in Figure 11, with the increase in MWCNT concentration, the GDH activity of maize leaves and roots first increased and then decreased, reaching the peak at the concentration of 800 mg·L^−1^. After treatment with low concentrations of MWCNTs (200 mg·L^−1^, 400 mg·L^−1^ and 800 mg·L^−1^), the GDH activity of maize leaves increased by 2.96%, 20.38% and 6.79%, respectively, compared with the control. After treatment with a high concentration of MWCNTs (1200 mg·L^−1^), the GDH activity of maize leaves decreased by 1.31%, but there was no significant difference. The GDH activity of maize roots increased by 1.37%, 26.01%, 7.51% and 1.58% compared with the control after treatment with MWCNTs of different concentrations, among which 200 mg·L^−1^ and 1200 mg·L^−1^ MWCNTs had no significant difference compared with the control, while the other treatments had significant differences. With the increase in nano-SiO_2_ concentration, the activity of GDH in maize leaves and roots increased first and then decreased, reaching the peak at 1500 mg·L^−1^ and 800 mg·L^−1^, respectively. After treatment with low concentrations of nano-SiO_2_ (150 mg·L^−1^, 800 mg·L^−1^ and 1500 mg·L^−1^), the GDH activity of maize leaves increased by 5.95%, 90.66% and 164.44%, respectively, compared with the control; the GDH activity of maize roots increased by 16.77%, 131.25% and 85.65%, respectively, compared with the control, and the difference was significant. After treatment with a high concentration of nano-SiO_2_ (2500 mg·L^−1^), the GDH activity of maize leaves decreased by 11.87% compared with the control, with a significant difference; the root activity of maize leaves decreased by 4.71%, but the difference was not significant. The effect of nano-SiO_2_ on the GDH activity of maize leaves and roots was stronger than that of MWCNTs (Figure 11).

## 3. Discussion

Nanocarbon has unique characteristics, such as high surface energy, and is important in promoting crop growth and development. Exogenous silicon can reduce the water loss of wheat leaves due to transpiration, and silicon can also induce the expression of aquaporin in sorghum roots [45,46,47]. It is reported that 100 mg·L^−1^ and 500 mg·L^−1^ nano-TiO_2_ treatments increased the dry weight of wheat roots, and the growth and fruit yield of cowpea plants treated with 500 mg·L^−1^ nano-TiO_2_ increased significantly [48,49]. Nanomaterials with lower concentrations can improve seedling vigor [19,50], dry matter quality [51], etc. In this study, different concentrations of MWCNTs and nano-SiO_2_ promoted the growth of maize seedlings, and had different degrees of promoting effects on maize plant height, root length, seedling dry and fresh weight, root–shoot ratio, etc. On the one hand, nano-materials have a negative charge on the surface and small particle size, which can promote plant growth. On the other hand, the special chemical structure of nanomaterials will also affect plant growth [52]. For example, the oxygen-containing functional groups and nitrogen-containing functional groups of nanomaterials can help plants absorb trace elements [53]. It may also be related to the hormone effect of MWCNTs and nano-SiO_2_. Nano-materials have the same regulatory effect as plant hormones, which can promote root elongation and seedling growth, and help seedlings adapt to the environment [54]. In addition, it may also be related to the strong antibacterial activity of nanomaterials. Some studies have shown that the antibacterial activity of nanomaterials is an essential reason for promoting plant growth [55].

An adverse living environment and damage to plants causes damage of plant cell membrane proteins, resulting in the exocytosis of cytoplasm, and ultimately the increase in the relative conductivity of plants [56]. Nano-ZnO significantly reduces the electrical conductivity of triticale plants under water-limited conditions and stabilizes and protects biofilm [57]. This study showed that lower concentrations of MWCNTs and nano-SiO_2_ decreased the relative conductivity of maize leaves. In comparison, higher concentrations of MWCNTs and nano-SiO_2_ increased the relative conductivity of maize leaves. The optimum concentration of MWCNTs is 800 mg·L^−1^, and the optimum concentration of nano-SiO_2_ is 1500 mg·L^−1^. It is suggested that lower concentrations of MWCNTs and nano-SiO_2_ improve the stability of maize plant cell membranes, which may be due to their enhanced nutrient absorption, root extension and the water status of the maize, while higher concentrations of MWCNTs and nano-SiO_2_ cause damage to maize plant leaves and damage the integrity of the leaf cell membrane. Relative water content (RWC) can judge the growth status of plants, and its value is vital for plant growth [58]. Research shows that nano-MgO can be absorbed by tomato roots and increase the relative water content of tomato plants [59]. Nano-Si alleviated the effect of salt stress on the water content of tomato leaves [47]. Nano-silica plays a positive role in the development of maize, especially in the up-regulation of relative water content, photosynthetic pigment, chlorophyll content and antioxidant enzyme activity of leaves [60]. Nanometer Fe_2_O_3_ of 100 and 500 mg·L^−1^ significantly increased the RWC of sorghum seedlings, maintained swelling pressure and improved salt tolerance [61]. Nano-TiO_2_ of different concentrations significantly increased the RWC of barley plants under different salt concentrations, and its high surface reaction activity may prolong the root pores or create new pores, resulting in increased water flow in the root [62]. Low-concentrations of nano-Se can increase the relative water content of leaves. Still, a high-concentration nano-Se will reduce the relative water content of Shanghaiqing leaves and affect its normal growth [63], indicating that the promotional effect of nano-materials on the relative water content of plants is limited by concentration, which is similar to the test results. The results of this study showed that different concentrations of MWCNTs and nano-SiO_2_ could increase the relative water content of maize seedling leaves, and with the increase in concentration, the promotional effect first increased and then weakened. Research shows that artificial nanoparticles, such as carbon nanotubes, increase water absorption by enhancing the expression of aquaporin, and positively impact broccoli’s growth under salinity stress [64]. This mechanism can also be used to explain the findings of this work. Superoxide anion (O_2_·^−^) is a kind of active oxygen free radical that can accelerate tissue membrane lipid peroxidation and promote plant aging. Hydrogen peroxide (H_2_O_2_) is a relatively stable signal molecule related to aging [65]. Low concentrations of H_2_O_2_ can protect plant cells, while high concentrations will produce toxic effects, so it is necessary to maintain normal ROS levels [66]. The research shows that nanoparticle treatment can induce the activity of specific antioxidant enzymes, reduce active oxygen levels, and improve the antioxidant status of seedlings [67]. Nano-titania treatment can significantly improve the activity of SOD, CAT and POD, reduce the accumulation of reactive oxygen radicals and MDA level, and maintain the stability of chloroplast membrane structure under light [21]. Nanometer iron oxide improves the antioxidant enzyme activity of watermelon and reduces the MDA concentration [68,69], thus improving the membrane’s integrity. In this experiment, MWCNTs and nano-SiO_2_ reduced the content of O_2_·^−^ and H_2_O_2_ in maize, which showed that MWCNTs and nano-SiO_2_ could prevent the damage of reactive oxygen species to cells, maintain the normal redox state of cells, maintain the complete structure of cells and slow down plant senescence. The enhanced activity of antioxidant enzymes can remove excess active oxygen species, thus reducing the degree of lipid peroxidation [23].

This study found that SWCNTs promoted the root growth of pumpkin and onion, but inhibited the root growth of tomatoes, while carrot and cabbage had no significant effect on root growth under SWCNT treatment [70]. Nano-ZnO and nano-carbon can promote the root growth of chickpea and wheat [16,71], respectively. The research shows that nano-CuO slows down the growth of wheat and mung bean seedlings, and their sensitivity is different. The decline of mung bean seedlings is significantly higher than that of wheat [72]. When the concentration of nano-CeO_2_ is 200 mg·L^−1^, it is beneficial to the growth of lettuce root [73], but when the concentration is more than 2000 mg·L^−1^, the root elongation is inhibited [74]. The above research shows that different kinds of nanomaterials have different effects on roots and different kinds of plants, and are affected by the concentration. The results of this experiment showed that the treatment of MWCNTs and nano-SiO_2_ with different concentrations promoted the growth of maize roots to different degrees. It shows that MWCNTs and nano-SiO2 at appropriate concentrations can promote plants’ root growth, thus improving plants’ ability to absorb water and nutrients, and is conducive to plant growth. Root activity reflects the growth of plants, the intensity of root tissue metabolism, and the ability to absorb and transport nutrients. Research shows that nano-material treatment can promote the growth of soybean, especially its root system, and enhance the vitality of plants’ root system [63]. Compared with high concentrations, low concentrations of nano-Fe_2_O_3_ have more obvious effects on root activity. When the concentration increases to 50 mg·L^−1^, the root activity of the sample treated with nano-Fe_2_O_3_ decreases sharply [68,75]. Studies have shown that carbon nanotubes can promote rice root activity at low concentrations, but may have toxic effects at high concentrations [76]. The results of this experiment showed that MWCNTs and nano-SiO_2_ both improved the root activity of maize seedlings, and the root activity showed a trend of first rising and then falling with the increase in its concentration, indicating that MWCNTs and nano-SiO_2_ can improve the root activity of the plant, improve the root absorption capacity of water and nutrients, and ultimately promote the growth of maize. Low-concentration MWCNTs and nano-SiO_2_ can improve the vitality of maize roots, which may be due to the fact that MWCNTs and nano-SiO_2_ promote the absorption of water and nutrients by plants. At the same time, because a large amount of energy consumption accompanies this process, plants can increase the absorption of nutrients and supplement energy demand by improving root vitality. High concentrations of MWCNTs and nano-SiO_2_ have a specific inhibitory effect on plant root activity, which may be due to the aggregation of nano-materials under high concentrations, resulting in the clogging of maize root pores, affecting the absorption of nutrients by root hair cells and resulting in a decline in root activity.

Phosphoenolpyruvate carboxylase (PEPC), phosphopyruvate dikinase (PPDK), malase (NADP-ME), malate dehydrogenase (NADP-MDH) and ribose-1,5-diphosphate carboxylase/oxidase (Rubisco) are five important photosynthetic enzymes involved in CO_2_ fixation of a plant’s dark reaction. The application of nanomaterials such as selenium and silicon dioxide has been proved to increase the growth and photosynthesis of plants [77]. Fe-based nanoparticles have chemical and structural magnetic effects on enzymes at different stages of photosynthesis. Adding low concentrations of molten iron to the culture medium can stimulate plant growth, while high concentrations of molten iron can inhibit plant growth [78]. The results of this experiment showed that the lower concentration of MWCNTs promoted the photosynthetic enzyme activity of maize leaves (PEPC, Rubisco, NADP-ME, NADP-MDH and PPDK), while the higher concentration of MWCNTs inhibited the photosynthetic enzyme activity of maize leaves. Lower concentrations of nano-SiO_2_ promoted the activities of NADP-MDH and PPDK enzymes in maize leaves, while higher concentrations of nano-SiO_2_ inhibited the activities of NADP-MDH and PPDK enzymes in maize leaves. Research shows that plant exposure to titanium dioxide nanoparticles promotes plant growth by increasing photosynthesis/light absorption [79,80]. Research shows that nanomaterials stimulate the increase in Rubisco, PEP carboxylase activity [81], the antioxidant enzyme system, etc. [82]. These effects may be the fundamental mechanism to promote crop growth and increase yield. It shows that the appropriate concentrations of MWCNTs and nano-SiO_2_ promote the stomatal opening of photosynthesis, improve the photosynthetic process of maize plants and promote plant growth by improving the activity of photosynthetic enzymes. PEPC is the key enzyme in the C4 pathway of photosynthesis in plants, and has great significance for the major metabolic pathways such as photosynthetic carbon assimilation. Rubisco activity is controlled by the rate of synthesis and degradation, and can regulate photosynthesis and photorespiration [83]. In this experiment, PEPC and Rubisco enzyme activities increased after treatment with low concentrations of MWCNTs, and decreased after treatment with a high concentration of MWCNTs. Different concentrations of nano-SiO_2_ increased after treatment, and its promotional effect showed a trend of increasing first and then decreasing with the increase in concentration. It shows that low concentrations of MWCNTs and nano-SiO_2_ promote the C_4_ dicarboxylic acid cycle of maize seedlings, improve the ability of maize seedlings to fix CO_2_, and lead to increased photosynthetic efficiency. However, high concentrations of MWCNTs can inhibit the PEPC activity of maize seedlings and affect the normal physiological and biochemical metabolism of maize, and the toxicity of MWCNTs on the PEPC activity of maize seedlings is greater than that of nano-SiO_2_. NADP-ME acts as a catalyst for the formation of pyruvate, NADPH and CO_2_ from malic acid and NADP+. The formed NADPH is used in the biosynthetic metabolic pathway to face the impact of abiotic stress [84]. NADP-MDH is mainly involved in the tricarboxylic acid cycle (TCA) and catalyzes the reversible conversion between malic acid and oxaloacetic acid [85]. In plants, MDH also participates in many other physiological and biochemical reactions, such as plant carbon fixation, nitrogen assimilation, fatty acid oxidation, etc. [86]. PPDK is a rate-limiting enzyme in the C4 pathway of plant photosynthesis. Its activity at a high level can promote nitrogen mobilization and increase protein content. The results of this study showed that low concentrations of MWCNTs and nano-SiO_2_ increased the activities of NADP-ME, NADP-MDH and PPDK, while high concentrations of MWCNTs and nano-SiO_2_ inhibited the activities of NADP-MDH and PPDK, but did not inhibit the activities of NADP-ME. The results showed that the sensitivity of different photosynthetic enzymes to MWCNTs and nano-SiO_2_ treatment was different. Low concentrations of MWCNTs and nano-SiO_2_ could promote the growth and development of maize, while high concentrations of MWCNTs and nano-SiO_2_ showed toxic effects.

Nitrogen metabolism is an important physiological process that can affect the metabolism and development of plants. Key enzymes such as glutamine synthetase (GS), glutamine synthetase (GOGAT), glutamic acid decarboxylase (GAD) and glutamic acid dehydrogenase (GDH) participate in this process and play an important catalytic and regulatory role. GDH and GS are the main enzymes of NH_4_^+^ assimilation and are also the important pathway of NH_4_^+^ assimilation. GOGAT participates in promoting the synthesis of amino acids, proteins and amino acids [87]. GDH is ubiquitous in plants and can catalyze NH_3_ and α-Ketoglutaric acid, forms of glutamic acid, and can also catalyze the oxidation of glutamic acid to release NH_3_. Research shows that nanoparticles can improve carbon and nitrogen metabolism, enhance light absorption, promote photosynthesis, and affect plant growth and development [88]. For example, nano-Si can enhance the key enzymes involved in glutamate synthesis, including glutamine synthetase (GS), glutamate dehydrogenase (GDH) and glutamate synthetase (GOGAT), which reveals the potential of nano-Si to regulate nitrogen transport through GS/GOGAT cycle [89]. Nano-TiO_2_ treatment can significantly improve the activity of nitrogen metabolism-related enzymes such as spinach NR, GDH, GS and GOGAT, and can promote the conversion of inorganic nitrogen to organic nitrogen (such as protein) [90]. Cerium dioxide enhances the activities of GOGAT, GS and glutamate dehydrogenase (GDH), the key enzymes of rice nitrogen assimilation, which is the reason for the increase in rice nitrogen content [91]. It has also been reported in soybeans, spinach and peanuts that metal-based nanomaterials enhance photosynthetic activity, nitrogen metabolism, and other physiological parameters [92]. In this study, the activities of GS, GOGAT, GAD and GDH in the leaves and roots of maize treated with MWCNTs and nano-SiO_2_ increased, which contributed to the accumulation of carbohydrates (sugar and starch), soluble protein and nitrogen in plants. These findings confirmed that MWCNTs and nano-SiO_2_ can promote plant growth by regulating the key enzymes of carbon and nitrogen metabolism, thus promoting the generation of carbohydrates and the utilization of nitrogen, and promoting plant growth [93]. The results of this experiment also showed that the content of pyruvate in leaves and roots of maize treated with MWCNTs and nano-SiO_2_ increased, which was consistent with the research results obtained on onions using nano-iron [94]. Pyruvate is an intermediate product of nitrogen metabolism, and the large increase in pyruvate content may indicate that nitrogen metabolism is more active. At the same time, it was found that the activities of GS, GOGAT, GAD and GDH in maize leaves and roots treated with high concentrations of MWCNTs and nano-SiO_2_ decreased, which may be due to the conversion of nitrogen metabolites in plants from anabolism to catabolism under the treatment of high concentrations of MWCNTs and nano-SiO_2_, or it may be due to its inhibition of nitrogen assimilation, thus affecting the synthesis of nitrogen metabolites. This means that high concentrations of MWCNTs and nano-SiO_2_ will interfere with plant nitrogen metabolism, and plant growth may be hindered.

## 4. Materials and Methods

### 4.1. Plant Materials and Growth Conditions

The tested maize variety was Fumin 985. The tested nanomaterials included two kinds; one comprised multi-walled carbon nanotubes (MWCNTs), and the other was nano-SiO_2_ (Table 3). Multi-walled carbon nanotubes were provided by Beijing Deke Island Gold Technology Co., Ltd., model: CNT105, length: 3–12 μm. Nano-silica was provided by Shanghai Waidian International Trade Co., Ltd., and the model was N20. The experiment was carried out in the laboratory of the Agricultural College of Northeast Agricultural University in 2021. We selected the seeds with full and healthy particles, soaked them in clean water for 24 h after disinfection treatment, and then evenly placed them in a disk with vermiculite, 80 seeds per disk, and watered them as appropriate. When the seedlings grew to 2 leaves and 1 heart, we selected the seedlings with consistent growth and moved them to a water tank containing 15 L of 1/2 Hoagland nutrient solution. We replaced the nutrient solution every 2 days, adjusted the pH value regularly, ventilated the device for 24 h and used a biological lamp to ensure 12 h of illumination. The conditions for seedling growth were: the photoperiod was 12/12 (day/night), the temperature was (28 ± 1) °C/(25 ± 1) °C day/night, the light intensity was 400 μmol·m^−2^·s^−1^ and the relative humidity was 60–70%. When the maize seedlings grew to three leaves and one heart, they were treated. We placed them in a water tank of 15 L nano-suspension of various concentrations prepared with 1/2 Hogland nutrient solution, set up a control group and applied the nutrient solution normally. Each test was processed three times.

### 4.2. Seedling Morphology and Dry–Fresh Weight

Five plants were randomly selected from each treatment, and the seedling height was measured with a ruler (plant height refers to the distance from the root neck to the top of the plant, where the top refers to the length of the tip of the maize leaf after straightening it). Then, we washed the seedlings with distilled water, absorbed the water, separated the roots and crowns of the seedlings and weighed the fresh weight. We then put the fresh sample in an oven at 105 °C for sterilization for 20 min, and then turned to 80 °C for drying to constant weight for 120 h. We weighed the dry weight and calculated the root–shoot ratio (fresh root mass/fresh seedling mass) [95].

### 4.3. Relative Water Content of Leaves

We selected five seedlings each time as test samples. The relative water content of leaves was determined by a drying and weighing method. After washing the collected leaves and absorbing the surface moisture, we weighed the fresh weight, and then immersed them in water for 24 h to fully absorb water to reach saturation. After absorbing the surface moisture, we weighed the saturated weight, and then put them in an oven at 105 °C for sterilization for 20 min, and then turned to 80 °C for drying to constant weight, and weighed the dry weight. We used the following formula to calculate the relative water content of leaves: RWC/% = [(fresh weight − dry weight)/(saturated weight − dry weight)] × 100% [95].

### 4.4. Relative Conductivity of Leaves

The relative conductivity (EC) of a leaf was measured by a conductivity meter (DDSJ-307F, Shanghai, China). We selected five seedlings each time as test samples. We immersed the sliced leaves in distilled water for 30 min, then washed them and absorbed the water, put them into a 10 mL glass tube, added 3 mL of distilled water and soaked them for 30 min, measured the conductivity (R1) at room temperature with a DDS-307 conductivity meter and then placed them in a boiling water bath for 5 min and measured the total conductivity (R2) after cooling to room temperature. We used the following formula to calculate: relative conductivity = R1/R2 × 100% [96].

### 4.5. Root Morphological Parameters

Five plants were randomly selected from each treatment. The roots were scanned with Epson Perfection root analyzer (V700, Beijing, China), the relevant root growth parameters, including total root length (TRL), total surface area (TSA), root volume (RV) and root mean diameter (RAD), were measured with a WinRhizo root analysis system and the number of lateral fresh roots and root tips were measured. Then, we put them in an oven at 95 °C for green removing for 20 min, then turned to 80 °C for drying to constant weight and measured the dry weight.

### 4.6. Root Activity

Preparation of standard curve: we put 2 mL of 0.4% triphenyl tetrazolium chloride (TTC) into a 100 mL volumetric flask, added a little sodium hydrosulfite, generated red triphenyl thyroid gland (TTF), used 95% ethanol to dilute to 100 mL and shook well. We took 0 mL, 0.5 mL, 1.0 mL, 1.5 mL, 2.0 mL, 2.5 mL and 3.0 mL of the above solutions into the test tube (≥15 mL), and then add 10 mL, 9.5 mL, 9 mL, 8.5 mL, 8 mL, 7.5 mL and 7 mL of 95% ethanol accordingly. Through the above steps, we obtained TTF 0 g, 40 g, 80 g, 120 g, 160 g, 200 g, 240 g and colorimetry at 485 nm.

Determination of root activity: we first weighed 0.2 g of maize seedling root (two plants), added it into 25 mL of test tube, added 5 mL of 0.4% TTC solution and 5 mL of 0.1 mol·L^−1^ phosphate-buffered solution (pH = 7.0), mixed the mixture, immersed the root tip and reacted at 37 °C for 1 h (dark reaction, sealing). Once the reaction time was up, we added 2 mL of 1 mol·L^−1^ H_2_SO_4_ to the test tube to terminate the reaction. We took out the root tip, dried the surface water of the root with absorbent paper and put it in a test tube. After adding 10 mL of methanol, we soaked it in an incubator for 6 h at a temperature of 30–40 °C. With methanol as the reference, we measured the absorbance at a wavelength of 485 nm.

### 4.7. DAB and NBT Staining Analysis

We used 3,3-diaminobenzidine (DAB) to dye hydrogen peroxide in maize leaves (five plants), referring to Orozco-Cardenas and other methods. Under the dark condition of 25 °C, we soaked the cut leaves in 1 mg·mL^−1^ DAB solution with a pH value of 3.8, and infiltrated them in vacuum for 8 h. Then, we immersed the leaves in boiling ethanol (96%) until the leaves decolored, cooled them and stored them in ethanol at room temperature and took photos [97]. The superoxide anion in maize leaves was stained with nitro blue tetrazole (NBT), referring to the methods of Romero-Puertas et al. We immersed the cut leaves (five plants) in 0.5 ng·mL^−1^ NBT solution containing 0.01 M PBS with a pH value of 6.4, and performed vacuum infiltration for more than 3 h until dark spots appeared. Then, we soaked the leaves in boiling ethanol until the leaves decolored, cooled them and stored them in ethanol at room temperature and took photos [98].

### 4.8. Photosynthetic Enzyme Activity of Leaves

The extraction and determination of the C4 enzyme refers to the methods of Johnson and Hatch, Sayre and Kennedy and Ashton [99,100]. We first took 0.5 g of fresh sample (three plants), ground it into powder with liquid nitrogen, then put it into a mortar containing 3 mL Tris-HCI buffer (pH = 7.5) and ground it in an ice bath until it was even. We centrifuged the extract obtained from the above procedure at 4 °C 10,000 r·min^−1^ for 20 min. The supernatant obtained above was used to analyze the activities of phosphoenolpyruvate carboxylase (PEPC), pyruvate phosphate double kinase (PPDK), malase (NADP-ME), fructose dehydrogenase (NADP-MDH) and ribose diphosphate carboxylase (Rubisco). PEPC enzyme activity was measured according to Gonzalez’s method [101], PPDK and Rubisco enzyme activity according to Hatch and Slack’s method [102] and NADP-ME and NADP-MDH enzyme activity according to Johnson’s method [99], and all were slightly adjusted. The ultraviolet and visible spectrophotometer used was a UV-2550 spectrophotometer from Shimadzu Company.

### 4.9. Pyruvic Acid Content

The extraction and determination of pyruvic acid (Pyr) refers to the method of Meng Deyi [103]. We first weighed 0.5 g of maize leaves (three plants) or roots, ground them evenly with 8% trichloroacetic acid in a mortar, and diluted them to 25 mL with trichloroacetic acid. We left it standing for 30 min, took 10 mL of homogenate and centrifuged it for 10 min at 12,000 rpm·min^−1^. The extracted supernatant was used to determine the content of pyruvate.

### 4.10. Enzyme Activity Related to Nitrogen Metabolism

For the extraction of glutamine synthetase (GS), refer to the method of Li Caifeng [104]. First, we weighed 1 g of leaves or roots (five plants), put them into a low-temperature treated mortar, ground them, added 2 mL of imidazole-hydrochloric acid (0.05 mmol·L^−1^, pH = 7.2) to extract buffer and used imidazole-hydrochloric acid to dilute them to 5 mL. Then, we centrifuged the test tube at 12,000 rpm·min^−1^ for 20 min, and the extracted supernatant was used to determine the enzyme activity. Refer to the methods summarized by Miflin and Lea [105]. The activity of glutamic acid synthase (GOGAT) was determined according to the method of Zheng Chaofeng et al. [106]. We took 1 g of leaves or roots (five plants), used a low-temperature treated mortar and pestle and added 2 mL of enzyme extraction buffer [100 mM KH_2_PO_4_ (pH7.5), 0.5 mM EDTA, 100 mM KCl, 0.5%(*v*/*v*) Triton X-100, 0.1%(*v*/*v*)] and 1 g of quartz sand to grind into a homogenate at low temperature. Then, we added 4 mL of extraction buffer solution and centrifuged it at 4 °C and 39,000 rpm·min^−1^ for 20 min, and determined the enzyme activity of the extracted supernatant. The activity of glutamic acid decarboxylase (GAD) and glutamic acid dehydrogenase (GDH) was determined with reference to Wang Lingxia [107], and the extraction method was the same as that of GOGAT. Only the extraction buffer was changed; we used 10 mL 50 mM sodium phosphate buffer (pH = 5.7) and 0.2 mol·L^−1^ Tris-HCl buffer 3.0 mL (pH = 8.2), and then the extracted supernatant was used to determine the enzyme activity.

### 4.11. Data Analysis

We used Excel 2016 to sort out the data and SPSS 22 statistical software to analyze the data; all data were tested for homogeneity of variance and then analyzed for one-way ANOVA (*p* < 0.05 is significant difference, *p* < 0.01 is extremely significant difference). Origin9.6 software was used for drawing.

## 5. Conclusions

The optimal concentration of MWCNTs is 800 mg·L^−1^, and the optimal concentration of nano-SiO_2_ is 1500 mg·L^−1^. MWCNTs and nano-SiO_2_ can promote the plant height, root length, seedling dry and fresh weight and root–shoot ratio of maize seedlings to different degrees. Under the appropriate concentration, the ability of maize to accumulate dry matter increased, the relative water content of leaves increased, the electrical conductivity of leaves decreased, the stability of cell membranes improved, and the water metabolism ability of maize seedlings increased. MWCNTs and nano-SiO_2_ both promoted the development of root morphology, increased root length, root surface area, average diameter, root volume and total root tip number, and improved root vitality, thus promoting the absorption and utilization of water and nutrients by plants. MWCNT and nano-SiO_2_ treatment reduced the content of O_2_·^−^ and H_2_O_2_ in maize leaves, and reduced the damage of reactive oxygen free radicals to cells. The effect is more significant under low concentration treatment, and it shows toxic effect under high concentration treatment. After treatment with MWCNTs and nano-SiO_2_, the activities of the key enzymes of photosynthesis in maize seedlings PEPC, Rubisco, NADP-ME, NADP-MDH and PPDK increased, the stomatal opening was promoted, the CO_2_ fixation efficiency was improved, the photosynthetic process of maize plants was improved and plant growth was promoted. MWCNTs and nano-SiO_2_ regulated the activities of enzymes related to nitrogen metabolism in maize seedlings. MWCNTs and nano-SiO_2_ increased the activities of the enzymes GS, GOGAT, GAD and GDH related to nitrogen metabolism in maize leaves and roots, increased the content of pyruvate, stabilized the transformation of various substances in the process of nitrogen metabolism and promoted the synthesis of carbohydrates and the utilization of nitrogen.

## Figures and Tables

**Table 1:** Effect of different concentrations of MWCNTs and nano-SiO_2_ on dry and fresh weight in maize.

Nanomaterials	Concentrations (mg·L^−1^)	Seedling Height (cm)	Seedling Fresh Weight (g)	Root Fresh Weight (g)	Root–Shoot Ratio	Root Dry Weight (g)	Shoot Dry Weight (g)
MWCNTs	0	32.113 ± 1.169 c	8.017 ± 0.803 c	2.321 ± 0.348 c	0.286 ± 0.016 b	0.527 ± 0.065 b	0.081 ± 0.010 c
	200	35.673 ± 1.116 b	9.264 ± 0.625 bc	3.049 ± 0.297 bc	0.328 ± 0.011 a	0.607 ± 0.024 ab	0.104 ± 0.012 bc
	400	42.850 ± 0.853 a	10.076 ± 0.301 ab	3.389 ± 0.166 ab	0.336 ± 0.007 a	0.651 ± 0.015 a	0.123 ± 0.011 ab
	800	45.670 ± 0.616 a	11.209 ± 0.374 a	3.933 ± 0.157 a	0.351 ± 0.002 a	0.702 ± 0.014 a	0.154 ± 0.009 a
	1200	37.487 ± 1.129 b	9.782 ± 0.375 abc	3.265 ± 0.206 ab	0.333 ± 0.011 a	0.625 ± 0.021 ab	0.113 ± 0.009 bc
Nano-SiO_2_	0	32.113 ± 1.169 d	8.017 ± 0.803 b	2.321 ± 0.348 c	0.286 ± 0.016 c	0.527 ± 0.065 b	0.081 ± 0.010 b
	150	37.020 ± 0.777 c	9.316 ± 0.625 b	2.782 ± 0.206 ab	0.298 ± 0.011 bc	0.621 ± 0.032 ab	0.109 ± 0.012 ab
	800	42.590 ± 1.187 b	10.051 ± 0.301 ab	3.227 ± 0.142 ab	0.321 ± 0.007 ab	0.683 ± 0.030 a	0.128 ± 0.007 a
	1500	47.123 ± 0.845 a	11.491 ± 0.374 a	3.778 ± 0.158 a	0.329 ± 0.002 a	0.756 ± 0.025 a	0.144 ± 0.012 a
	2500	39.223 ± 0.908 bc	9.529 ± 0.295 ab	2.918 ± 0.194 bc	0.306 ± 0.011 abc	0.631 ± 0.032 ab	0.117 ± 0.011 ab

Note: Data are expressed as mean ± standard deviation. Different letters within the same column indicate significant difference at 5% level.

**Table 2:** Effect of different concentrations of MWCNTs and nano-SiO_2_ on seedling root morphology in maize.

Nanomaterials	Concentrations (mg·L^−1^)	Root Length (cm)	Root Surface Area (cm^2^)	Average Diameter of the Root (mm)	Root Volume (cm^3^)	Number of Root Tips
MWCNTs	0	11.19 ± 0.64 c	3.69 ± 0.36 b	1.17 ± 0.17 abc	0.12 ± 0.02 b	60.33 ± 8.95 c
	200	11.48 ± 0.66 c	3.91 ± 0.38 b	1.48 ± 0.21 a	0.14 ± 0.02 b	101.67 ± 11.46 c
	400	18.59 ± 0.61 b	4.81 ± 0.32 ab	0.86 ± 0.05 bc	0.18 ± 0.02 ab	468.33 ± 42.44 b
	800	22.16 ± 0.92 a	5.78 ± 0.19 a	0.71 ± 0.06 c	0.22 ± 0.02 a	810.67 ± 35.48 a
	1200	13.11 ± 0.60 c	4.36 ± 0.37 b	1.30 ± 0.24 ab	0.15 ± 0.03 ab	129.67 ± 14.68 c
nano-SiO_2_	0	11.19 ± 0.64 d	3.69 ± 0.36 c	1.17 ± 0.17 a	0.12 ± 0.02 b	60.33 ± 8.95 c
	150	13.50 ± 0.73 cd	4.20 ± 0.51 bc	1.24 ± 0.15 a	0.12 ± 0.02 b	124.67 ± 26.89 bc
	800	15.78 ± 0.68 bc	4.95 ± 0.22 b	0.77 ± 0.09 ab	0.18 ± 0.01 ab	176.00 ± 39.72 b
	1500	25.29 ± 1.50 a	6.20 ± 0.35 a	0.67 ± 0.06 b	0.23 ± 0.02 a	785.33 ± 15.93 a
	2500	17.66 ± 0.73 b	5.30 ± 0.23 ab	1.10 ± 0.15 ab	0.13 ± 0.02 b	146.00 ± 23.80 bc

Note: Data are expressed as mean ± standard deviation. Different letters within the same column indicate significant difference at 5% level.

**Table 3:** Physicochemical properties of MWCNTs and nano-SiO_2_.

Name	CAS Number	Diameter (nm)	Purity (wt%)	Specific Surface Area (m^2^·g^−1^)	Appearance
MWCNTs	308068-56-6	20–30	>98.0	>233	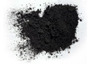
nano-SiO_2_	60676-86-0	10–20	>99.8	>200	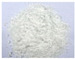

## Data Availability

Not applicable.
